# Progress in the study of the mechanism of ferroptosis in coronary heart disease and clinical intervention strategies

**DOI:** 10.3389/fcvm.2025.1545231

**Published:** 2025-04-16

**Authors:** Yingzhi Liu, Zixuan Yu, Yuwen Lu, Yue Liu, Lingli Chen, Jie Li

**Affiliations:** ^1^ Hunan Key Laboratory of TCM Diagnostics, Hunan University of Chinese Medicine, Changsha, China; ^2^ Hunan Key Laboratory of Pathogeny Biology of Integrated Chinese and Western Medicine, Hunan University of Chinese Medicine, Changsha, China

**Keywords:** coronary heart disease, ferroptosis, iron metabolism, mechanism, clinical intervention strategies

## Abstract

Coronary heart disease (CHD), a serious cardiovascular condition with complex and diverse pathogenesis, has recently seen increased attention to the role of ferroptosis—a novel iron-dependent form of programmed cell death. This review synthesizes current research on ferroptosis mechanisms in CHD and emerging clinical intervention strategies. Ferroptosis is characterized by dysregulated iron metabolism, lipid peroxidation, and reactive oxygen species (ROS) accumulation, processes intimately linked to CHD pathophysiology. Under ischemic and hypoxic conditions commonly seen in coronary artery disease (CAD), cardiomyocytes become particularly susceptible to ferroptosis, resulting in cellular dysfunction and diminished cardiac performance. Mechanistic studies have revealed that altered expression of iron metabolism-related proteins (including GPX4, FTH1, TfR1, and HO-1), accumulation of lipid peroxidation products, and disruption of antioxidant defense systems (particularly the Nrf2/GPX4 pathway) are central to ferroptosis progression in cardiac tissue. Clinically, both specific ferroptosis inhibitors (such as Ferrostatin-1) and traditional medicine components (such as Puerarin) have emerged as promising therapeutic candidates, showing cardioprotective effects in experimental models. However, research into ferroptosis mechanisms in CHD remains in its early stages, with significant questions regarding its relationship with other cell death pathways and the clinical efficacy of ferroptosis-targeting interventions requiring further investigation. Future research directions should include in-depth mechanistic exploration and the development of more effective, safer clinical interventions targeting the ferroptosis pathway in cardiovascular disease.

## 1 Introduction

Coronary heart disease (CHD) represents a significant threat to global health, characterized by complex and multifactorial pathogenesis (1). Epidemiological studies have highlighted the substantial burden of CHD across diverse populations, with various risk factors contributing to its development, including type 2 diabetes, hypertension, and hyperhomocysteinemia (2–4). In recent years, ferroptosis—a distinctive iron-dependent programmed cell death mechanism—has emerged as a focus of intense scientific investigation. This process, triggered by lipid peroxidation and excessive reactive oxygen species (ROS) accumulation, offers a novel perspective on the pathophysiology and potential therapeutic approaches for CHD (5, 6).

The pathophysiological significance of ferroptosis in cardiovascular disease stems from its multifaceted involvement in abnormal iron metabolism, lipid peroxidation, and ROS accumulation—processes fundamental to the development of coronary artery disease (CAD). Cardiomyocytes in CAD patients exhibit particular vulnerability to ferroptosis under adverse conditions such as ischemia and hypoxia, leading to cellular dysfunction and compromised cardiac performance (7). Recent investigations have revealed that ferroptosis involves alterations in iron metabolism regulatory proteins (e.g., GPX4, TfR1), accumulation of lipid peroxidation products, and dysregulation of antioxidant systems, particularly the nuclear factor E2-related factor 2 (Nrf2)/glutathione peroxidase 4 (GPX4) pathway (8, 9). These insights have not only enhanced our understanding of CHD pathophysiology but have also established a theoretical foundation for developing ferroptosis-targeted therapeutic strategies. Consequently, ferroptosis inhibitors and antioxidants have become focal points in clinical intervention research (9). For instance, Ferrostatin-1, a specific ferroptosis inhibitor, has demonstrated significant attenuation of adriamycin-induced cardiac injury and heart failure in murine models, suggesting a promising drug target for CAD treatment. Additionally, traditional Chinese medicines and their bioactive components have shown promising anti-ferroptotic effects through multi-target and multi-pathway mechanisms (10). Studies have demonstrated that compounds such as Puerarin can reduce cardiomyocyte death by inhibiting ferroptosis, presenting novel options for CHD prevention and treatment (11, 12).

However, research on ferroptosis mechanisms in CHD remains in its nascent stages, with numerous crucial questions awaiting resolution. The specific mechanisms by which ferroptosis contributes to CHD pathophysiology require further elucidation, particularly regarding its interrelationship with other cell death pathways (e.g., apoptosis, necrosis). Moreover, additional clinical trials are essential to validate the efficacy and safety of ferroptosis-targeting interventions. Therefore, future research should focus on deepening our understanding of ferroptosis mechanisms in CAD and developing more effective and safer clinical intervention strategies to enhance CHD management.

Ferroptosis represents a distinct iron-dependent form of programmed cell death that differs fundamentally from apoptosis, necrosis, and autophagy in both mechanism and morphological characteristics. Unlike apoptosis, which involves caspase activation and nuclear fragmentation, ferroptosis is initiated by iron accumulation and lipid peroxidation, resulting in membrane rupture without caspase involvement (13). Necrosis, typically resulting from severe injury, manifests as uncontrolled cell death with cellular content leakage, whereas ferroptosis proceeds as a regulated process driven by oxidative stress and iron overload, primarily through ROS-induced lipid damage (14). Autophagy, a cellular survival mechanism under stress conditions, can lead to cell death if excessive; however, ferroptosis is specifically induced by iron dysregulation and lipid peroxidation (15, 16). In CHD, ferroptosis contributes to myocardial injury through disruption of iron homeostasis and promotion of oxidative damage, highlighting its potential as a therapeutic target (15).

This review aims to comprehensively analyze recent advances in understanding ferroptosis mechanisms in CHD and evaluate emerging clinical intervention strategies. By synthesizing the role of ferroptosis in CHD pathophysiology and identifying potential therapeutic targets, we seek to provide novel insights and approaches for the prevention and treatment of this prevalent cardiovascular condition.

## 2 Overview of the basis of coronary heart disease and ferroptosis

### 2.1 Pathophysiology of coronary heart disease

CHD is fundamentally a cardiovascular disorder precipitated by atherosclerosis of the coronary arteries. The pathophysiological cascade encompasses several critical processes: atherosclerosis development, thrombosis formation, and myocardial ischemia. Coronary atherosclerosis initiates with endothelial damage to the vascular intima, followed by a sequence of events including lipid deposition, vascular smooth muscle cell proliferation, and extracellular matrix synthesis, collectively resulting in arterial wall thickening and progressive luminal narrowing (17). As atherosclerotic plaques develop and advance, coronary blood flow becomes increasingly restricted, particularly during periods of elevated myocardial oxygen demand such as physical exertion or emotional stress. This restriction precipitates myocardial ischemia which, when sustained, can trigger cardiomyocyte injury, apoptosis, or necrosis, ultimately compromising cardiac function.

Thrombosis represents another critical aspect in acute CHD manifestations. When atherosclerotic plaques rupture or endothelial integrity is compromised, rapid platelet aggregation occurs, activating the coagulation cascade and culminating in thrombus formation. These thrombi can further occlude coronary arteries, leading to acute myocardial infarction (AMI). Such events can precipitate extensive cardiomyocyte death, resulting in pronounced cardiac function deterioration and, in severe cases, life-threatening complications (18).

Myocardial ischemia constitutes the predominant mechanism underlying CHD pathophysiology and serves as the central pathological process. It initiates a cascade of deleterious changes, including impaired energy metabolism, enhanced ROS production, and intracellular calcium overload. These factors collectively contribute to myocardial cell injury and death, affecting both systolic and diastolic cardiac function while promoting adverse myocardial remodeling and eventual heart failure progression (19).

### 2.2 Definition and characterization of ferroptosis

**Figure 1:**
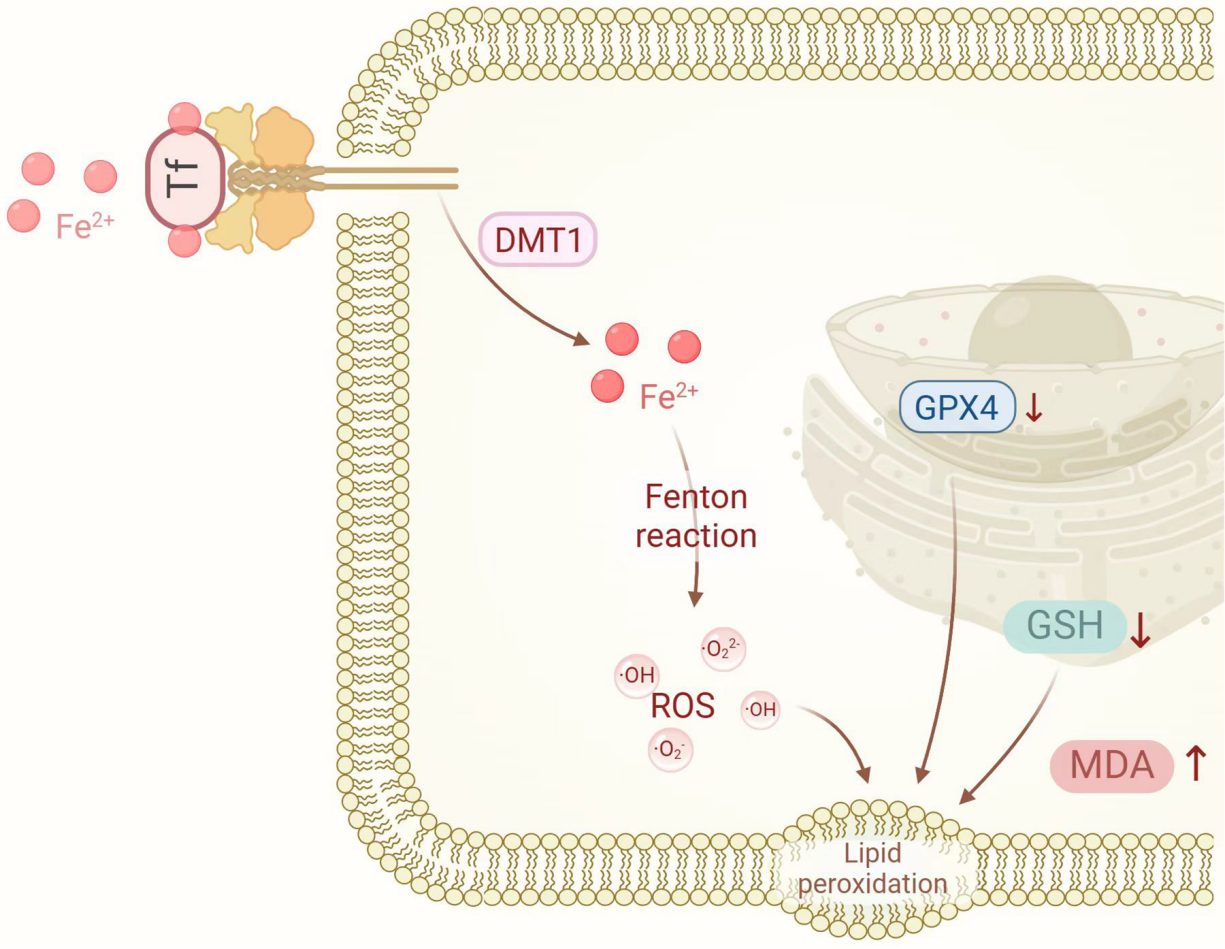
The mechanisms of ferroptosis. DMT1, divalent metal transporter 1; GPX4, glutathione peroxidase 4; GSH, glutathione; MDA, malondialdehyde; ROS, reactive oxygen species. Created in BioRender. Liu, Y. (2025) https://BioRender.com/wpn71vi.

The mechanisms of ferroptosis are illustrated in Figure 1.

Ferroptosis, first characterized by the Brent R. Stockwell laboratory in 2012, represents an iron-dependent, non-apoptotic cell death modality distinguished by intracellular iron accumulation and lipid peroxidation, culminating in cell membrane disruption and cellular demise (13). The mechanisms governing ferroptosis primarily involve iron metabolism dysregulation, lipid peroxidation, glutathione (GSH) depletion, and reduced GPX4 activity.

Disrupted iron homeostasis constitutes a primary trigger for ferroptosis initiation. Elevated intracellular iron levels facilitate the generation of ROS through Fenton chemistry, subsequently initiating lipid peroxidation reactions (20). Lipid peroxidation represents the central process in ferroptosis and establishes a destructive cycle wherein interactions between iron ions and lipid peroxidation products exacerbate oxidative damage to cellular membranes. Concurrently, GSH—a critical intracellular antioxidant—becomes depleted, compromising the cell's capacity to neutralize ROS and lipid peroxidation products, thereby accelerating ferroptotic progression (21). GPX4, a GSH-dependent antioxidant enzyme, plays a pivotal role in reducing lipid peroxidation products and protecting cell membranes from oxidative insult. When GPX4 activity diminishes, lipid peroxidation proceeds unimpeded, consequently triggering ferroptosis (22).

Compared with other forms of cell death such as apoptosis, necrosis, and autophagy, ferroptosis exhibits distinct molecular mechanisms and biological features (Table 1):

(1) Iron Dependence: Ferroptosis is uniquely triggered by elevated intracellular iron levels, differentiating it from other cell death modes. Apoptosis, in contrast, involves the activation of caspase family proteins and morphological changes, such as nuclear condensation and fragmentation. Apoptosis plays a key role in tissue homeostasis and the removal of damaged cells. Necrosis is a non-programmed form of cell death caused by external factors, characterized by membrane rupture and the release of cellular contents. Autophagy, on the other hand, involves the degradation of damaged organelles or proteins through autophagosomes, and while it can be protective under stress conditions, excessive autophagy can also lead to cell death.
(2) Lipid Peroxidation: Ferroptosis is marked by significant lipid peroxidation, with products like malondialdehyde (MDA) serving as biomarkers. This phenomenon is rarely observed in other forms of cell death.
(3) PUFAs in Membranes: Polyunsaturated fatty acids (PUFAs) on cell membranes are oxidized and destroyed during ferroptosis, leading to loss of cell membrane integrity and cell death.
(4) Complex Ferroptosis involves multiple signaling pathways and molecular regulatory networks, including iron metabolism, lipid metabolism, antioxidant system, etc., which reflects the complexity of the ferroptosis pathway and provides a potential target for clinical intervention in related diseases (23–25).

**Table 1:** Overview of cell death types.

Cell death types	Ferroptosis	Apoptosis	Necroptosis	Autophagy
Definition	Iron-dependent programmed cell death	Apoptosis, an orderly process of cell suicide	Necroptosis, a type of programmed necrosis	Autophagy, a process of cell self-digestion and degradation
Morphological character (Cytomembrane)	Impaired integrity, potential rupture	Maintains integrity, forms apoptotic bodies	Rupture, loss of integrity	No significant changes
Morphological character (Cytoplasm)	Iron deposition and lipid peroxidation	Cytoplasmic condensation, organelle aggregation	Cytoplasmic swelling, organelle dissolution	Autophagic vacuoles appear, engulfing cytoplasmic components
Morphological character (Nucleus)	Minimal changes or mild condensation	Chromatin condensation, nuclear fragmentation	Nuclear dissolution, chromatin dispersion	Minimal changes or Mild deformation
Biochemical characteristic	Increased lipid peroxidation, elevated iron levels	DNA fragmentation, Caspase activation	RIPK1/3 activation, MLKL phosphorylation	ATG family protein activation, lysosomal enzyme release
Core regulatory molecule	GPX4, HSPB1, NOX4, Nrf2, p53, RAS, Slc7a11, TfR1, VDAC2/3, etc.	Initiator caspases (caspase-2, -8, -9, -10, -12), executioner caspases (caspase-3, -6, -7), IAP, PUMA, p53, Bax, Bak, Bcl-2, Bcl-XL, and other pro-apoptotic Bcl-2 family proteins	RIP1, RIP3, MLKL	AMPK, ATG (ATG-5, -7, -8, -10, -12, and -16l, etc.), Beclin1, mTOR, p62, ULK1/2, Vps34, etc.
Agonist and inducer	DPI7/10, erastin, FINO2, RSL3, SOR, etc.	Actinomycin D, camptothecin, Cytc, cycloheximide, CD95l, TNF-α	FasL, IFNs, MLKL agonists, RIPK3 agonists, TRAIL, TNF-α, etc.	Brefeldin A, thapsigargin, tunicamycin, rapamycin and its derivatives, EBSS, carbamazepine, L-690,330, lithium chloride, PP242, AZD8055, N-Acetyl-D-sphingosine, xestospongin, torin1, WAY600, etc.
Inhibitor	DFO, DFP, ciclopirox, α-tocopherol, Fer1, Lip1, trolox, XJB-5-131, troglitazone, rosiglitazone, pioglitazone, TEMPO, PHOXNO, selenium compounds, AZD8055, INK128, torin1, etc.	Z-VAD-FMK, Q-VD-OPh, ABT-737, ABT-263, rapamycin, LY294002, necrosulfonamide, necrostatin-1 and its racemates, GSK-872, etc.	Necrostatin-1, necrosulfonamide, GSK'872, GSK'843, etc.	Hydroxychloroquine, bafilomycin A1, 3-MA, wortmannin, LY294002, etc.

### 2.3 Relationship between iron metabolism and coronary heart disease

A significant interrelationship exists between iron metabolism and CHD, with dysregulation of iron homeostasis potentially influencing both the risk and progression of CHD. Iron represents an essential trace element in human physiology, participating in crucial processes including hemoglobin synthesis, oxygen transport, and diverse enzymatic reactions. However, both iron excess and deficiency can adversely impact cardiovascular health (26).

Elevated iron levels may generate substantial ROS through Fenton reactions, triggering oxidative stress and lipid peroxidation, thereby exacerbating coronary atherosclerosis development and progression. Additionally, iron overload may enhance platelet aggregation and thrombosis, increasing the risk of acute coronary events (26–28). Several epidemiological studies have established positive correlations between excessive iron levels and CHD morbidity and mortality. For instance, a meta-analysis demonstrated that elevated serum ferritin levels were significantly associated with increased cardiovascular event risk in general populations (29).

Conversely, iron deficiency can similarly exert negative effects on cardiovascular function. Iron deficiency anemia, a prevalent condition, diminishes hemoglobin's oxygen-carrying capacity, resulting in tissue hypoxia. Chronic iron insufficiency may cause irreversible damage to vital organs including the heart, cerebral vasculature, and kidneys. Furthermore, iron deficiency anemia may disrupt lipid metabolism and vascular endothelial function, further elevating CHD risk. Iron redox activity, while integral to numerous physiological processes, can catalyze ROS formation and lipid peroxidation, directly linking iron dysregulation to CHD development (15). Therefore, maintaining balanced iron levels is crucial for cardiovascular health and CHD prevention.

In clinical practice, iron status assessment typically involves measuring serum ferritin, transferrin saturation, and related parameters, enabling the development of personalized iron supplementation or chelation treatment protocols based on individual patient conditions. Furthermore, advanced research into ferroptosis mechanisms in CHD may identify novel targets and strategies for CHD prevention and treatment, potentially mitigating myocardial injury by inhibiting ferroptosis-related signaling pathways or enhancing antioxidant capacity, ultimately improving cardiac function and quality of life for CHD patients.

## 3 Mechanisms of ferroptosis in coronary heart disease

**Figure 2:**
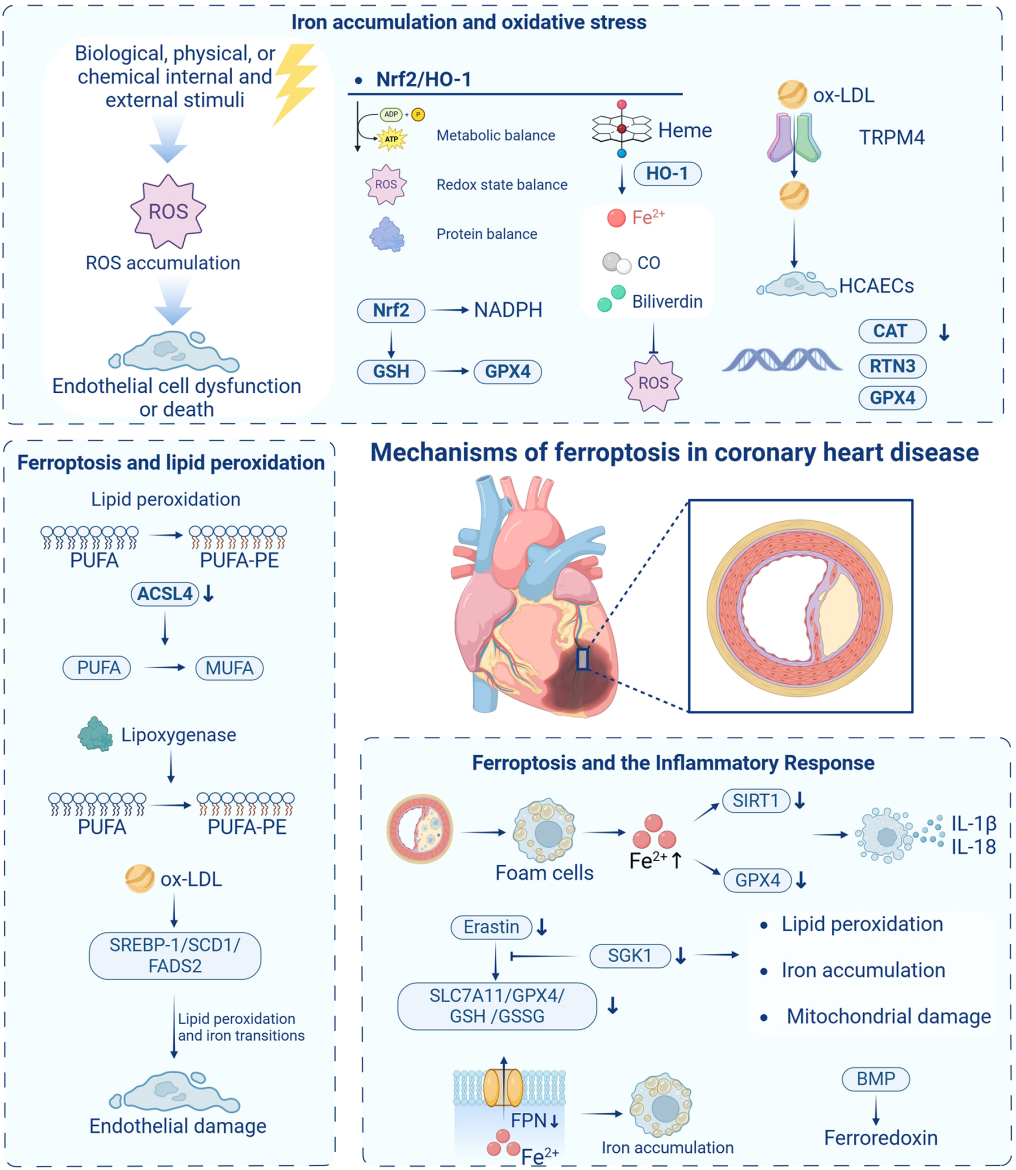
The mechanisms of ferroptosis in coronary heart disease. ROS, reactive oxygen species; Nrf2, nuclear factor E2-related factor 2; HO-1, heme oxygenase-1; NADPH, nicotinamide adenine dinucleotide phosphate; GSH, glutathione; CO, carbon monoxide; ox-LDL, oxidized low-density lipoprotein; TRPM4, transient receptor potential melastatin 4; HCAECs, human coronary artery endothelial cells; CAT, catalase; RTN3, reticulon 3; PUFA, polyunsaturated fatty acids; PUFA-PE, polyunsaturated fatty acid phospholipids; ACSL4, acyl coenzyme A synthetase long-chain family member 4; MUFA, monounsaturated fatty acid acyls; SREBP-1, sterol regulatory element-binding protein 1; SCD1, stearoyl-CoA desaturase 1; FADS2, fatty acid desaturase 2; SIRT1, silent information regulator 1; GPX4, glutathione peroxidase 4; SGK1, serum/glucocorticoid-regulated kinase 1; Slc7a11, solute carrier family 7 member 11; GSSG, oxidized glutathione; FPN, ferroportin-membrane iron transport protein; BMP, bone morphogenetic protein. Created in BioRender. Liu, Y. (2025) https://BioRender.com/wpn71vi.

The mechanisms of ferroptosis in coronary heart disease are illustrated in Figure 2.

### 3.1 Iron accumulation and oxidative stress

Oxidative stress represents a fundamental mechanism in CHD pathogenesis and serves as a critical trigger for cellular ferroptosis. When the organism encounters internal or external stressors—biological, physical, or chemical—that disrupt cellular homeostasis, ROS production may exceed elimination capacity, resulting in ROS accumulation. Excessive ROS levels can induce cellular damage, initiating a cascade of effects that potentially culminate in tissue and organ injury (30). While ROS can function as second messengers regulating cellular reproduction, growth, differentiation, and signaling when maintained at optimal concentrations and dynamic equilibrium, exceeding critical ROS thresholds induces oxidative stress, leading to DNA damage, protein denaturation, and lipid peroxidation—key factors in CHD development. Consequently, targeted inhibition of cellular ROS generation represents a promising therapeutic approach for CHD management.

#### 3.1.1 Impact of ROS on cellular function and atherosclerosis

Elevated ROS can induce endothelial cell dysfunction or death, disrupting the vascular barrier and leading to vasoconstrictive and vasodilatory dysfunction, vascular smooth muscle cell proliferation and migration, inflammatory responses, and thrombosis—processes intimately associated with various cardiovascular disorders, including atherosclerosis, hypertension, and CHD (31). Therefore, targeted inhibition of *in vivo* ROS generation represents a promising therapeutic strategy for CHD treatment.

#### 3.1.2 Role of Nrf2/HO-1 pathway in ferroptosis and antioxidant defense

The Nrf2/heme oxygenase-1 (HO-1) pathway plays a central role in oxidative stress-induced ferroptosis. Nuclear factor E2-related factor 2 (Nrf2), a key transcription factor and endogenous antioxidant, maintains cellular metabolic homeostasis, redox state, and protein homeostasis. Under physiological conditions, the Neh2 structural domain of Nrf2 binds to Kelch-like ECH-associated protein 1 (Keap1) in the cytoplasm, facilitating Nrf2 ubiquitination and subsequent degradation (32). However, under oxidative stress conditions, Nrf2 dissociates from Keap1 and translocates to the nucleus, where it activates various antioxidant genes, including HO-1, protecting cells from oxidative damage.

#### 3.1.3 TRPM4 channels and endothelial dysfunction in atherosclerosis

Ye et al. (33) demonstrated that transient receptor potential melastatin 4 (TRPM4) channels significantly contribute to oxidized low-density lipoprotein (ox-LDL)-induced dysfunction in human coronary artery endothelial cells (HCAECs). TRPM4 knockdown substantially reduced cellular inflammation, oxidative stress, and lipid peroxidation, thereby improving endothelial function and potentially modulating atherosclerosis progression. Additionally, gene expression studies examining reticulon 3 (RTN3), GPX4, and catalase (CAT) in CAD patients have identified novel molecular markers, enhancing our understanding of ferroptosis in CAD development (34).

#### 3.1.4 Nrf2/Slc7a11/GPX4 axis and myocardial injury

In ischemia-reperfusion injury (IRI), a well-established model for myocardial injury, ozone pre-treatment mitigates myocardial ferroptosis by activating the Nrf2/solute carrier family 7 member 11 (Slc7a11)/GPX4 axis. Using *in vivo* and ex vivo ischemia-reperfusion models, ozone treatment was shown to reduce myocardial injury by regulating ferroptosis-related proteins and transcription factors, particularly enhancing Nrf2 nuclear translocation, thereby exerting cardioprotective effects (35). This indicates that Nrf2 activation could serve as an effective strategy to attenuate oxidative stress and ferroptosis in myocardial injury.

#### 3.1.5 Mechanisms of ferroptosis in myocardial ischemia-reperfusion injury

In pathological conditions such as myocardial ischemia-reperfusion injury, the Nrf2/Keap1 pathway becomes dysregulated, leading to the dissociation of Nrf2 from Keap1 and its subsequent translocation into the nucleus. This endogenous protective mechanism primarily inhibits oxygen radical generation by upregulating antioxidant factors such as HO-1, thereby mitigating oxidative stress. Research by Wang et al. (36) demonstrated that key signaling pathways—including the Nrf2-Keap1 pathway, p53, and Hippo pathway—contribute to the initiation and progression of atherosclerosis by modulating the expression and activity of ferroptosis-related proteins. Importantly, Nrf2 target genes function beyond NADPH regeneration and GSH-dependent reduction of thioredoxin and peroxiredoxin; they also regulate iron metabolism and GSH synthesis, highlighting their multifaceted roles in controlling cellular ferroptosis. HO-1, a critical antioxidant enzyme, enhances cellular defense against oxidative damage by catalyzing heme breakdown to produce Fe²⁺, carbon monoxide (CO), and biliverdin (35). However, paradoxically, excessive Fe²⁺ accumulation can trigger intracellular iron overload, promoting ferroptosis. This complex interplay within the Nrf2/HO-1 pathway remains under active investigation, as it represents a double-edged sword that can be either protective or harmful depending on the precise balance of iron and ROS levels within the cell (37).

#### 3.1.6 Role of NADPH oxidase in ferroptosis

NOX, a widely distributed membrane protein in humans, represents the only enzyme capable of directly catalyzing ROS generation (38). During ischemia-reperfusion injury, NOX overexpression and enhanced activity contribute to ROS generation, subsequently promoting cellular ferroptosis. NOX-catalyzed superoxide anion production is converted to hydrogen peroxide by superoxide dismutase (SOD), further affecting cellular function through membrane oxidation of cysteine (Cys). Disruption of the NOX enzyme system severely impairs GSH production, thereby exacerbating cellular ferroptosis risk.

#### 3.1.7 Voltage-dependent anion channels in ferroptosis and oxidative stress

Voltage-dependent anion channels (VDACs), membrane pore proteins located in the outer mitochondrial membrane, facilitate ion and metabolite transport in eukaryotic cells (39). Erastin, a ferroptosis inducer, binds to VDAC2 and VDAC3 on the outer mitochondrial membrane, inducing mitochondrial dysfunction and oxidant release, ultimately triggering ferroptosis in tumor cells. Additionally, upregulated Ras-RAF-MEK pathway expression may enhance cancer cell sensitivity to ferroptosis by modulating VDAC2/3 function and increasing ROS generation (40). Thus, VDACs represent an emerging focus in ferroptosis research, while oxidative stress in CHD pathogenesis is further evidenced by low-density lipoprotein (LDL) conversion to oxidized LDL. Oxidized LDLs play crucial roles in initiating and amplifying inflammatory responses at lesion sites, promoting leukocyte recruitment, and contributing to atherosclerosis progression through vascular smooth muscle cell activation and reduced nitric oxide bioavailability.

### 3.2 Ferroptosis and lipid peroxidation

#### 3.2.1 Definition and mechanism of lipid peroxidation

Lipid peroxidation represents a process triggered by excess ROS, involving oxidative generation of polyunsaturated fatty acid phospholipids (PUFA-PE). PUFA-PE refers to cell or organelle membrane phospholipids containing PUFAs, which exhibit particular vulnerability to oxidative damage. This process is closely linked to imbalances between oxidative and antioxidant systems, with homeostatic maintenance being essential for cellular integrity (36). During peroxidation, multiple hydroperoxide (ROOH) components interact with proteins and DNA, damaging these biomolecules. Phospholipid hydroperoxides (PLOOHs) execute a critical role in ferroptosis under peroxidizing conditions, catalyzing and generating complex products including oxygen radicals, while ROS trigger cellular damage by promoting membrane PUFA peroxidation (41).

#### 3.2.2 Relationship between lipid peroxidation and ferroptosis

Lipid peroxidation manifests as a continuous process of free radical production and reaction—a chain reaction that severely compromises cell membrane integrity, ultimately leading to organelle and/or cell membrane rupture (42). PUFA-PE production depends on coordinated action between acyl coenzyme A synthetase long-chain family member 4 (ACSL4) and lysophospholipid acyltransferase 3 (LPCAT3), which collectively activate and incorporate PUFAs (e.g., docosahexaenoic acid, arachidonic acid) into membrane lipids (43).

Bioinformatic analyses of CHD-related datasets have identified differentially expressed genes (DEGs) associated with immune and inflammatory responses. Among these, serum/glucocorticoid-regulated kinase 1 (SGK1) expression patterns in circulating endothelial cells showed consistency between healthy individuals and CHD patients. *in vitro* experiments demonstrated that SGK1 knockdown attenuated erastin-induced downregulation of Slc7a11, GPX4, GSH, and oxidized glutathione (GSSG), while reducing lipid peroxidation, iron accumulation, and mitochondrial damage upregulation, suggesting SGK1's critical role in CHD-associated ferroptosis (44).

#### 3.2.3 Role of lipid peroxidation in coronary heart disease

ACSL4, a key upstream regulator of lipid peroxidation, has garnered significant attention for its role in ferroptosis. ACSL4 facilitates long-chain unsaturated fatty acid (e.g., arachidonic acid 20:4, adrenoic acid 22:4) incorporation into phospholipids through coordinated action with lysophospholipid acyltransferases. Genetic deletion or pharmacological inhibition of ACSL4 significantly shifts long-chain phospholipid PUFA composition toward short-chain monounsaturated fatty acid acyls (MUFA) in phospholipids. Notably, ACSL4-deficient cells evade ferroptosis, continuing to proliferate for months following GPX4 knockdown. Consequently, inhibiting ACSL4 expression represents an important direction in ferroptosis research (43).

The final step of PUFA-PE peroxidation requires lipoxygenase catalysis, suggesting a potential role for these enzymes in ferroptosis induction. Studies demonstrate that pharmacological lipoxygenase inhibition can prevent ferroptosis. Additionally, arachidonic acid-15-lipoxygenase (ALOX15) knockdown or treatment with the lipoxygenase inhibitor baicalein has shown protective effects against ischemic brain damage in murine models (5, 45). However, lipoxygenase primarily contributes to cysteine starvation-induced ferroptosis rather than GPX4 deficiency-induced ferroptosis. Therefore, the precise mechanisms through which lipoxygenases contribute to ferroptosis, particularly in GPX4 inhibition contexts, require further investigation (15).

#### 3.2.4 Therapeutic targets and experimental evidence

Lipid peroxidation not only affects biological membrane fluidity and membrane protein function, impairing cellular activity, but its products also form reactive aldehyde end products, including 4-hydroxynonenal (4-HNE) and MDA. 4-HNE represents a highly reactive lipid peroxidation byproduct that induces cellular damage by interacting with proteins, DNA, and other cellular components through iron- and oxygen-mediated chain-breaking reactions (7). These reactive aldehydes can attack additional intracellular targets, including proteins and DNA, extending initial membrane damage to other macromolecules and cellular regions.

The transient receptor potential channel TRPM4 has been shown to promote ox-LDL-induced dysfunction in HCAECs through ferroptosis mechanisms (33). Research demonstrated elevated TRPM4 expression in ox-LDL-treated HCAECs, while TRPM4 knockdown reduced intracellular iron levels and ferroptosis-related protein expression (33).

Additionally, reduced sterol regulatory element-binding protein 1 (SREBP-1) expression—a key transcription factor in lipid and cholesterol metabolism—is associated with stable CAD development. During ox-LDL-induced endothelial injury, SREBP-1/stearoyl-CoA desaturase 1 (SCD1)/fatty acid desaturase 2 (FADS2) expression decreased and participated in ferroptosis processes. Mechanistically, ox-LDL induces lipid peroxidation by inhibiting the SREBP-1/SCD1/FADS2-mediated lipid biosynthesis pathway, leading to endothelial damage and ultimately inducing ferroptosis (46). This highlights the potential significance of the SREBP-1/SCD1/FADS2 pathway in regulating lipid metabolism and ferroptosis in atherosclerosis contexts.

### 3.3 Ferroptosis and the inflammatory response

The discovery of ferroptosis may explain iron accumulation's biological effects. Recent research found that the silent information regulator 1 (SIRT1) autophag*y* axis in foam cells inhibits excess iron-induced ferroptosis. Su et al. (47) demonstrated that excess iron induced ferroptosis in foam cells by adding exogenous ferric ammonium citrate to THP-1 cells. Excess iron inhibited the SIRT1-autophag*y* axis in foam cells and decreased GPX4 expression, collectively inducing foam cell ferroptosis. Ferroptotic foam cells release substantial inflammatory mediators, including IL-1β and IL-18, which belong to the IL-1 cytokine family and undergo caspase-1-mediated activation (48, 49). Earlier studies have demonstrated that IL-1β and IL-18 not only promote atherosclerosis progression but also contribute to advanced plaque instability and myocardial infarction (50). Thus, macrophage ferroptosis and macrophage-derived foam cell ferroptosis persist throughout plaque formation and progression to advanced unstable plaques. SGK1 promotes ferroptosis via the NEDD4l/NF-*κ*B pathway in CHD. *in vitro* experiments showed that SGK1 knockdown alleviated erastin-induced downregulation of Slc7a11, GPX4, GSH, and GSSG, while reducing lipid peroxidation, iron accumulation, and mitochondrial damage upregulation, suggesting that SGK1 modulates ferroptosis processes through inflammatory pathways in CHD (44). In SARS-CoV-2 infection cases, epinephrine (Epi) administration unexpectedly attenuated cytokine storms, decreased ferritin levels, and suppressed ferroptosis. SARS-CoV-2 infection was observed to cause pathological changes including acute respiratory distress syndrome, elevated pro-inflammatory mediator release, dysregulated iron metabolism, and iron overload, with Epi administration significantly improving cardiac status in these patients (51).

Zheng et al. (31) highlighted that endothelial cell, comprising the single-layer epithelium of the vascular system, play crucial roles in maintaining intravascular homeostasis. Endothelial dysfunction or death can precipitate various pathological conditions, including vascular barrier disruption and vasoconstrictive/vasodilatory dysfunction. ROS overproduction-induced oxidative stress represents a principal mechanism of endothelial cell death, with ROS activating multiple pathways leading to endothelial cell demise. Regulating macrophage ferroptosis and macrophage-derived foam cells has emerged as a novel therapeutic target for coronary atherosclerotic heart disease. Iron is essential for macrophage ferroptosis, with the ferroportin-membrane iron transport protein (FPN) axis representing the primary pathway regulating macrophage iron levels (52). Iron modulators degrade FPN (the sole iron export protein) on macrophage surfaces, increasing intracellular iron content. Inhibiting hepcidin expression enables macrophages to excrete excess iron and cholesterol, thereby reducing foam cell formation and atherosclerosis progression (52). Bone morphogenetic protein (BMP) stimulates ferredoxin expression under conditions of increased iron levels or inflammation. Similarly, BMP inhibition significantly delays atherosclerosis and vascular calcification (53). While current research confirms that macrophage ferroptosis promotes atherosclerosis progression and that hepcidin influences atherosclerotic processes by regulating iron content, the precise relationship between macrophage ferroptosis and hepcidin remains unestablished. Whether hepcidin can influence atherosclerosis development by regulating macrophage ferroptosis represents an emerging research focus.

## 4 Clinical testing and assessment related to ferroptosis

### 4.1 Biomarkers

#### 4.1.1 Definition and mechanism of ferroptosis biomarkers

Ferroptosis, as a novel form of programmed cell death, involves complex mechanisms and multiple biomolecules and metabolic pathways (54, 55). The identification and detection of biomarkers associated with ferroptosis activity are crucial for understanding its mechanisms in CAD and evaluating the efficacy of clinical interventions (6, 22). Lipid peroxidation, a principal feature of ferroptosis, generates products such as MDA, 4-hydroxyhexenal, and 4-HNE that serve as valuable indicators for assessing the extent of lipid peroxidation and ferroptosis (14, 56). In a one-year follow-up study, elevated serum MDA levels were identified as independent predictors of mortality in CAD patients, supporting the hypothesis that lipid peroxidation contributes to both the development and severity of heart disease (57).

#### 4.1.2 Markers related to iron metabolism and ferroptosis

Iron-related protein levels represent important biomarkers for assessing ferroptosis activity. Abnormal iron metabolism is a significant trigger for ferroptosis, with iron-related proteins including transferrin receptor protein 1 (TfR1), ferritin (FTH and FTL), and iron export proteins playing essential roles in iron homeostasis. For instance, TfR1 mediates cellular iron uptake, and its upregulation increases iron acquisition, thereby exacerbating ferroptosis. Ferritin serves as the primary iron storage protein, with its levels reflecting intracellular iron content. Additionally, Wen et al. conducted a systematic review and meta-analysis to evaluate the diagnostic and prognostic value of serum ferritin in predicting intravenous immunoglobulin (IVIG) resistance and coronary artery lesions in patients with Kawasaki disease (KD). They found that serum ferritin demonstrated relatively high sensitivity, specificity, and area under the receiver operating characteristic curve (AUC) values for KD diagnosis and exhibited strong prognostic value for predicting IVIG resistance and coronary artery lesions (58).

#### 4.1.3 Novel biomarkers for ferroptosis in CAD

Recent years have witnessed the identification of novel ferroptosis biomarkers in CAD. Lai et al. (59) identified xanthurenic acid (XA) as a new biomarker demonstrating high sensitivity and specificity in myocardial infarction patients. Mechanistic investigations revealed that elevated XA levels induced myocardial injury by promoting myocardial apoptosis and ferroptosis. Inhibition of kynurenine 3-monooxygenase (KMO) significantly suppressed XA elevation and attenuated both oxygen-glucose deprivation (OGD)-induced cardiomyocyte injury and ligation-induced myocardial ischemia injury, suggesting XA's potential utility as a ferroptosis marker in CAD.

In advanced atherosclerosis stages, prostaglandin endoperoxide synthase 2 (PTGS2), ACSL4, caspase-1, and NOD-like receptor protein 3 (NLRP3) expression is upregulated, while GPX4 is downregulated. These markers' expression correlates with atherosclerosis severity, with PTGS2 demonstrating potential as a key gene. This suggests that ferroptosis, in conjunction with pyroptosis (*via* NLRP3), may regulate atherosclerosis progression, offering insights into potential therapeutic targets for CAD (6).

#### 4.1.4 Non-coding RNAs as biomarkers for ferroptosis in CAD

Beyond protein-based biomarkers, non-coding RNAs have emerged as ferroptosis biomarkers relevant to CAD. Previous research identified that lncRNA lnc-MRGPRF-6:1 promotes ox-LDL-induced macrophage ferroptosis by inhibiting GPX4. Lnc-MRGPRF-6:1 expression is elevated in CAD patients and strongly associates with macrophage-mediated inflammation. Knockdown of lnc-MRGPRF-6:1 attenuated ox-LDL-induced macrophage ferroptosis, while overexpression exacerbated this process. Furthermore, lnc-MRGPRF-6:1 showed high expression in monocyte-derived macrophages from CAD patients and exhibited negative correlation with GPX4 expression (60).

#### 4.1.5 Ferroptosis-related gene biomarkers in atherosclerosis and myocardial infarction

Moreover, Liu et al. identified potential ferroptosis-related gene biomarkers in atherosclerosis and analyzed their correlation with immune infiltration characteristics using bioinformatic approaches. They screened six differentially expressed ferroptosis-related genes (IL6, ANGPTL7, CDKN1A, AKR1C3, NOX4, and VLDLR) and constructed a random forest model based on these genes, which demonstrated strong diagnostic performance in the validation dataset, highlighting the close relationship between ferroptosis and immunity in atherosclerosis pathogenesis (61). Wu et al. (57) identified ferroptosis-related genes and immune infiltration biomarkers in AMI through comprehensive analysis, finding that PIK3CA represented a robust diagnostic biomarker for AMI. They constructed an immune-related regulator*y* axis involving XIST and OIP5-AS1/miR-216a/PIK3CA, which may play a critical role in regulating ferroptosis during AMI progression.

### 4.2 Imaging

#### 4.2.1 Introduction to imaging techniques for ferroptosis detection

Studies combining imaging techniques such as MRI and magnetic nanoparticles for *in vivo* ferroptosis observation initially emerged in oncology research, where ferroptosis modulation represents a novel treatment approach that potentially overcomes resistance to conventional cancer therapies. Magnetic nanoparticles promote ferroptosis by supplying ferrous ions while enabling targeted ferroptosis cancer nanomedicines and concurrent MRI diagnostics, offering image-guided ferroptosis cancer nanotherapeutics (62).

#### 4.2.2 Advancements in imaging ferroptosis in myocardial ischemia

Recent technological advancements have facilitated basic and clinical studies combining magnetic particles with imaging tools such as MRI to determine ferroptosis levels in ischemic myocardium. Researchers in China synthesized an optical-magnetic multimodal probe targeting TfR1, a ferroptosis-specific molecular marker, and employed molecular imaging technology to achieve dynamic visualization and monitoring of tanshinone IIA efficacy in treating myocardial ischemia/reperfusion injury and elucidating ferroptosis pathway regulatory mechanisms. This non-invasive visualization technology not only validated tanshinone IIA's efficacy and mechanism but also established an effective imaging tool for detecting ferroptosis levels in CAD (63).

#### 4.2.3 Multimodal imaging for myocardial ischemia-reperfusion injury

A 2024 study introduced a novel multimodal imaging platform for non-invasive *in vivo* identification of myocardial ischemia-reperfusion cardiac lesion markers. This platform utilized superparamagnetic cubic iron oxide nanoparticles (SCIO NPs) targeting magnetic particle imaging (feMPI) for ferroptosis detection with TfR1 as a characteristic biomarker. Results demonstrated that feMPI signal intensity linearly correlated with myocardial infarction percentage in the MI/R mouse model, enabling quantitative assessment of cardiac lesion extent. Notably, feMPI detected cardiac injury approximately 48 h earlier than conventional clinical detection methods, providing a powerful tool for MI/R-induced ferroptosis investigation and offering insights for molecular imaging and drug development targeting ferroptosis-related therapies (63).

#### 4.2.4 FeMRI for early detection of cardiotoxicity

Additionally, for anticancer drug-associated acute cardiac/renal injury, Fantian et al. developed a probe utilizing redox-active Fe (II) as a chemical target to visualize ferroptosis processes. This approach demonstrated high feasibility for early *in vivo* diagnosis of ACI/AKI, detecting these conditions at least 24 and 48 hours earlier than standard clinical assays for ACI and AKI, respectively. Moreover, feMRI provided imaging evidence for different ferroptosis-targeting drugs' mechanisms of action, whether by blocking lipid peroxidation or depleting iron ions. This study presented a simple, effective feMRI strategy for early assessment of anticancer drug-induced ACI/AKI, potentially offering new approaches for detecting and evaluating ferroptosis in CHD (64).

#### 4.2.5 Inhibition of ferroptosis in sepsis-induced myocardial injury

In sepsis-induced myocardial injury, ferroptosis inhibition may represent a novel therapeutic strategy. Previous study established a LPS-induced murine sepsis model (65) and found that small, biocompatible, MRI-visible melanin nanoparticles (MMPP) significantly improved cardiac function and attenuated myocardial structural disorders. Mechanistic studies suggested that MMPP protects myocardium from sepsis-induced injury by inhibiting the iron accumulation-induced ferroptosis signaling pathway. Furthermore, *in vitro* experiments demonstrated that MMPP inhibited cardiomyocyte death by attenuating oxidative stress and inflammatory responses while maintaining mitochondrial homeostasis. These findings suggest that MMPP protects the heart from sepsis-induced myocardial injury by inhibiting ferroptosis and inflammatory responses, potentially providing novel approaches for future therapies.

## 5 Progress in research on clinical intervention strategies

Table 2 summarizes key intervention strategies and their mechanisms, helping to provide a clearer understanding of how each strategy targets ferroptosis and its potential application in cardiovascular diseases and other related conditions (12, 37).

**Table 2:** Clinical intervention strategies for ferroptosis.

Intervention	Mechanism of action	Stage of research/Clinical application
Iron Chelators (Deferoxamine, Deferiprone, Deferasirox)	Iron chelators reduce intracellular Fe3+ levels, preventing its conversion to Fe2+, and inhibit lipid peroxidation by tightly binding to ferric ions.	Used clinically for iron overload (thalassemia); experimental in cardiovascular diseases
Rapamycin	Inhibits the mTOR complex, reducing transferrin receptor expression, decreasing cellular iron content, and activating the Irp1/2 system.	Experimental; studied for cardioprotection in iron-overload conditions
Cornflowerin-3-O-glucoside (C3G)	Inhibits ferroptosis induced by RSL3, reduces Fe2+ levels, and regulates the expression of TfR1 and FTH1, as well as modulates NCOA4 expression.	Preclinical; used in reducing oxidative stress and ferroptosis
Baicalein	Acts as a potent iron chelator and inhibits Fe2+ production in Erastin-induced ferroptosis. It also enhances the expression of Nrf2 and the activity of HO-1.	Clinical studies on cardioprotective effects; antioxidant and anti-ferroptosis activity
Mucuna pruriens	Reduces ROS and RNS levels, thereby mitigating oxidative stress and preventing ferroptosis.	Preclinical; antioxidant properties
Danshensu	A potent ROS scavenger, improves antioxidant activity by enhancing SOD, catalase, GPX4, and HO-1, protecting the heart through antioxidant effects.	Clinical and experimental cardiovascular and cerebrovascular protection
Probucol	Increases Nrf2 and GPX4 protein expression, reducing oxidative stress and lipid peroxidation in cardiomyocytes, with a lipid-lowering effect.	Clinical use for hypercholesterolemia; potential for atherosclerosis treatment
Ferroptosis Suppressor Protein 1 (FSP1)	Reduces CoQ to CoQH2, inhibiting lipid peroxidation and ferroptosis by trapping lipid peroxidation free radicals.	Preclinical; emerging therapeutic strategy in cancer and ferroptosis research
GCH1-BH4 Pathway	GCH1, a rate-limiting enzyme in BH4 synthesis, promotes CoQH2 production and phospholipids containing PUFA tails, contributing to ferroptosis defense by trapping lipid radicals.	Early research; needs further investigation of its role in subcellular compartments
Yiqi Fuxin Injection (YQFM)	Inhibits ferroptosis in SIC by affecting the xCT/GPX4 axis.	Clinical and animal studies for SIC treatment
Cagliflozin	Inhibits ferroptosis by regulating inflammation and ferroptosis through the AMPK pathway, providing cardioprotective effects.	Preclinical studies for cardiotoxicity prevention
Baicalein-Peptide Nanofibers	Targets angiotensin II type I receptor to deliver baicalein directly to damaged cardiomyocytes, inhibiting ferroptosis and protecting against doxorubicin-induced cardiotoxicity.	Experimental studies for cancer chemotherapy protection
SGLT2 Inhibitors (Empagliflozin)	Attenuates cardiotoxicity from trastuzumab by inhibiting DNA damage and ferroptosis, protecting mitochondrial integrity.	Clinical research on cardiotoxicity in cancer patients
NCOA4 and SFXN1 Targeting	Inhibits NCOA4-mediated ferritinophagy and SFXN1 activation to prevent ferroptosis in LPS-induced myocardial injury, improving cardiac function and survival.	Preclinical; new targets for the prevention of ferroptosis in heart disease

### 5.1 Iron chelators and iron modifiers

Iron chelators and modifiers effectively inhibit cellular ferroptosis by regulating the expression and activity of iron transporters and storage proteins, including transferrin and its receptor (TfR1), ceruloplasmin, ferroportin, divalent metal transporter 1 (DMT1), and other membrane iron transport proteins (24, 26). Additionally, they can reduce the expression and activity of key iron uptake proteins such as transferrin receptor, divalent metal transporter 1 (DMT1), and zinc-iron transporters, while also modulating critical regulatory factors including iron-regulated proteins and hypoxia-inducible factor 1 (27). Intracellular Fe^2+^ exists in the form of a labile iron pool and binds to low molecular weight compounds such as GSH (20). GPX4 inactivation but also promotes the formation of GSH-Fe complexes. These complexes are delivered to ferritin via the ferric iron molecular chaperone poly(C)-binding protein 1 (PCBP1), exacerbating labile iron availability and ferroptosis progression.

To reduce intracellular Fe^3+^ levels and prevent its conversion to Fe^2+^, iron chelators are widely employed. Among these, deferoxamine, deferiprone, and deferasirox are most commonly used. These compounds reduce oxidative stress catalysis by tightly binding ferric ions, ultimately inhibiting lipid peroxidation processes. While iron chelators have demonstrated efficacy in treating transfusion-associated iron overload in thalassemia patients, their cardiovascular applications remain predominantly experimental (24, 66). Rapamycin, an inhibitor of mammalian target of rapamycin (mTOR) complex 1, exhibits cardioprotective effects in ferroptotic cells, where mTOR overexpression protects against iron-overload-induced cell death, a process exacerbated by mTOR deficiency (67, 68). Rapamycin exerts anti-ferroptotic effects by decreasing transferrin receptor expression, reducing cellular iron content, and activating the iron-regulated protein-1/2 (Irp1/2) system, potentially compensating for transferrin downregulation to maintain iron homeostasis (69).

Cyanidin-3-O-glucoside (C3G), an anthocyanin analog widely present in purple and red fruits and vegetables, effectively inhibits RSL3-promoted ferroptosis. C3G not only attenuates cellular and tissue oxidative stress but also reduces Fe^2+^ levels, inhibits nuclear receptor coactivator 4 (NCOA4) expression, and concurrently regulates transferrin receptor protein 1 (TfR1) downregulation and ferritin heavy chain 1 (FTH1) upregulation. Research has demonstrated that NCOA4 knockdown reduces intracellular iron levels and lipid peroxidation, thereby attenuating zinc nanoparticle-induced cell death (70). Additionally, baicalein, an active component from the traditional Chinese medicine Scutellaria baicalensis, functions as a potent iron chelator and inhibits erastin-induced Fe^2+^ production under normal cellular conditions, potentially playing a key role in regulating organismal iron homeostasis and inhibiting Fenton reactions (71).

### 5.2 Antioxidant therapy

Oxidative stress can induce varying degrees of damage to organisms. Mitigating pro-oxidant factors or enhancing antioxidant mechanisms can effectively alleviate these detrimental effects. An emerging strategy to address ROS accumulation toxicity involves developing or characterizing small molecules that specifically scavenge ROS at particular intracellular sites.

#### 5.2.1 Key antioxidant pathways in ferroptosis regulation

In critical oxidative stress response pathways, Nrf2/HO-1 signaling is paramount, with Nrf2 serving as a key endogenous antioxidant factor. Certain pharmaceuticals enhance antioxidant effects by upregulating Nrf2 expression. Additional antioxidant pathways, including the GTP cyclohydrolase 1 (GCH-1)-tetrahydrobiopterin (BH4) pathway and the ferroptosis suppressor protein 1 (FSP1)-ubiquinone (CoQ)-NADPH pathway, also play vital roles in ferroptosis regulation.

#### 5.2.2 Key antioxidant pathways in ferroptosis regulation

Vitexin, a flavonoid monomer extracted from hawthorn leaves, mung bean peels, and other plants, demonstrates pharmacological effects in reducing ROS and RNS levels. Che et al. (72) demonstrated, using a rat left coronary artery ligation model, that vitexin reduced MDA levels in cardiomyocytes while increasing SOD and NADPH activities, thereby inhibiting lipid peroxidation, preventing cardiomyocyte ferroptosis, and improving cardiac function. Lu et al. (73) further confirmed in animal experiments that vitexin enhanced Nrf2 expression and HO-1 activity, reduced cellular ROS production, and consequently inhibited ferroptosis.

Baicalein, another important flavonoid, demonstrates significant cardioprotective effects through activation of the Nrf2 signaling pathway, thereby attenuating the oxidative stress damage caused by tert-butyl hydroperoxide in H9C2 cardiomyocytes. In the presence of specific ferroptosis-inducing agents, baicalein exhibited superior anti-ferroptosis activity compared to established ferroptosis inhibitors such as ferrostatin-1, liproxstatin-1, desferrioxamine mesylate, and β-mercaptoethanol (71, 74). Another study (75) showed that danshensu, a potent ROS scavenger, possesses significant pharmacological activities including antioxidant, anti-inflammatory, antiplatelet aggregation, and antitumor effects, and has a wide range of cardiovascular and cerebrovascular protective properties. It enhances the activity of endogenous antioxidants such as SOD, catalase, GPX4, and HO-1 and protects the heart through these antioxidant effects.

#### 5.2.3 Trace elements and pharmacological agents in antioxidant therapy

Recent studies of trace elements selenium and vitamin E in CHD have demonstrated their ability to improve cellular lipid metabolism and counteract lipid peroxidation. Probucol, a lipid-lowering agent, also exhibits antioxidant properties that reduce intracellular oxidative stress and maintain normal cellular redox state by upregulating Nrf2 and GPX4 protein expression, thereby enhancing cardiomyocyte antioxidant capacity. Its lipid-improving efficacy has been utilized in hypercholesterolemia treatment. Probucol's antioxidant effects make it a potential alternative therapy for improving atherosclerotic disease and effectively preventing carotid atherosclerosis progression in hypercholesterolemic patients (76). However, due to its moderate cholesterol-lowering effect, QT interval prolongation, and insufficient clinical evidence for cardioprotective effects, probucol has not achieved widespread clinical adoption.

#### 5.2.4 Ferroptosis suppressor protein 1 and coenzyme Q10 pathway

FSP1 possesses NAD(P)H oxidoreductase activity and reduces CoQ to ubiquinol (CoQH2), which inhibits lipid peroxidation and ferroptosis by trapping lipid peroxidation free radicals (77, 78). This suggests that FSP1 exerts anti-ferroptotic effects by generating a non-mitochondrial CoQH2 pool. Notably, while CoQ is primarily synthesized in mitochondria, it is also detected in non-mitochondrial membranes, including the plasma membrane. The precise origin of non-mitochondrial CoQ utilized by FSP1 for ferroptosis defense requires further investigation. FSP1 has received considerable attention in cancer research, with numerous studies (77, 78) examining its expression and function in various tumor cells, finding close associations with apoptosis and antitumor effects.

Besides the FSP1-CoQ pathway, recent evidence has revealed an alternative ferroptosis defense mechanism involving dihydroorotate dehydrogenase (DHODH) and coenzyme Q10. As demonstrated by Mao et al. (2021), DHODH contributes to ferroptosis resistance by generating an intracellular CoQH2 pool independent of the FSP1 pathway (79). However, significant controversy exists regarding the relative contributions of these parallel pathways in different cellular contexts. Some studies suggest that DHODH activity is particularly crucial under conditions of GSH depletion, while others propose that the pathway's significance varies according to tissue type and metabolic state (80, 81). Moreover, the potential crosstalk between the DHODH/CoQ10 and FSP1 pathways remains debated, with some evidence suggesting competitive and others indicating cooperative interactions. Further research is needed to elucidate the precise regulatory mechanisms governing the DHODH/CoQ10 pathway in myocardial ischemia-reperfusion injury and coronary heart disease progression.

In the GCH1-BH4 pathway, BH4 functions as an antioxidant capable of trapping peroxidized lipid radicals, with GCH1 serving as the rate-limiting enzyme in BH4 biosynthesis. Additionally, GCH1 promotes CoQH2 production and phospholipids (PLs) containing two PUFA tails, which provide ferroptosis defense. However, the specific subcellular compartments in which the GCH1-BH4 pathway operates require further investigation (14).

Yiqi Fuxin injection (YQFM), comprising ginseng, maitake, and schisandra, ameliorates sepsis-induced cardiomyopathy (SIC) by inhibiting ferroptosis. Both cellular and animal studies have demonstrated that YQFM significantly inhibits myocardial ferroptosis and the xCT/GPX4 axis, resulting in SIC amelioration (82). Canagliflozin significantly inhibits inflammatory factor expression, including cyclooxygenase-2 (COX-2) and inducible nitric oxide synthase, exerting cardioprotective effects by regulating inflammation and ferroptosis through the AMP-activated protein kinase (AMPK) pathway (83).

#### 5.2.5 Traditional Chinese medicine in antioxidant therapy

Traditional Chinese medicinal preparations containing active ingredients such as ginsenosides and Panax ginseng saponins have demonstrated significant protective effects in CHD. These saponin components act through multiple targets and pathways, including anti-oxidative stress, anti-inflammatory, and anti-apoptotic mechanisms. Studies have also revealed that saponin components can regulate ferroptosis-related protein expression, thereby attenuating cardiomyocyte ferroptosis and providing novel therapeutic options for CHD management (84).

### 5.3 Ferroptosis-targeted therapeutic strategies in coronary heart disease

#### 5.3.1 Inhibiting ferroptosis in coronary atherosclerotic plaques

The pathogenesis of coronary atherosclerotic plaques involves iron accumulation and lipid peroxidation—hallmarks of ferroptosis. Zhou et al. (6) identified PTGS2 as a hub gene in human coronary artery atherosclerosis, with increased expression correlating directly with plaque instability and lesion severity. This coronary-specific finding demonstrates that ferroptosis is not merely associated with general cardiovascular pathology but plays a central role in coronary plaque development.

Clinical studies have confirmed elevated iron content specifically in coronary plaques from patients with acute coronary syndrome compared to stable coronary artery disease (27). The ferroptosis inhibitor ferrostatin-1 selectively reduced macrophage death in coronary lesions and decreased plaque vulnerability in experimental models of coronary atherosclerosis (85). These coronary-specific interventions demonstrate that targeted ferroptosis inhibition represents a promising approach for preventing plaque progression and rupture in coronary heart disease.

#### 5.3.2 Protection against ferroptosis in ischemic myocardium after coronary events

Following coronary artery occlusion, the resulting myocardial ischemia-reperfusion injury involves extensive ferroptotic cardiomyocyte death. Wu et al. (57) analyzed ferroptosis-related gene expression patterns specifically in myocardium following coronary occlusion, revealing an immune-ferroptosis axis that influences post-infarction remodeling. Importantly, these mechanisms were found to be distinct from those in other forms of cardiovascular injury, highlighting coronary-specific pathways. Yang et al. (63) developed a coronary-targeted approach using nanoparticles that specifically detect ferroptotic regions in ischemic myocardium following coronary events. This method enabled the visualization of the injured myocardial area supplied by occluded coronary arteries with significantly greater sensitivity than conventional techniques. The same targeting mechanism allowed for selective delivery of ferroptosis inhibitors to the affected coronary territory, demonstrating superior cardioprotection compared to systemic therapy in experimental coronary occlusion models.

#### 5.3.3 Ferroptosis in coronary microvascular dysfunction

Coronary microvascular dysfunction, a distinct pathological entity within the spectrum of coronary heart disease, involves significant ferroptotic endothelial cell death. Ye et al. (33) demonstrated that TRPM4 channels promote oxidized LDL-induced dysfunction specifically in human coronary artery endothelial cells through mechanisms involving ferroptosis. This finding establishes a direct link between ferroptosis and coronary microvascular pathology.

Clinical trials evaluating SGLT2 inhibitors in patients with coronary microvascular dysfunction have shown improvements in coronary flow reserve through mechanisms involving ferroptosis inhibition (86). Myocardial perfusion imaging in these patients revealed enhanced microcirculatory function specifically in the coronary circulation, without significant effects on other vascular beds. These findings suggest that modulating ferroptosis pathways offers targeted benefits for the coronary microvasculature, addressing a specific component of coronary heart disease that often persists despite successful epicardial coronary intervention.

## 6 Limitation and future research priorities

Despite the promising potential of ferroptosis-targeting therapies, several limitations currently hinder their translation into clinical practice. One significant challenge is the incomplete understanding of ferroptosis pathways, particularly their complexity and the multitude of interacting molecules involved. This complexity complicates the development of precise and effective therapies that can specifically modulate ferroptosis without disrupting other essential cellular processes. Additionally, while preclinical studies have demonstrated encouraging results, most research remains in early stages, and the lack of reliable biomarkers for monitoring ferroptosis complicates clinical application. Furthermore, potential adverse effects, such as off-target toxicity of ferroptosis inhibitors or activators, require thorough evaluation. Inhibition of key ferroptosis regulators like acyl coenzyme ACSL4, GPX4, and Nrf2 may lead to unintended consequences, including disruptions to cellular homeostasis, thereby posing potential risks to therapeutic safety. Finally, translating these therapies into clinical settings remains challenging due to issues such as drug delivery optimization, variability in individual responses, and the necessity for personalized treatment strategies.

To address these limitations, future research should focus on several key areas. First, a more comprehensive understanding of ferroptosis mechanisms in different cell types, tissues, and pathological conditions is critical. Identifying the specific triggers and molecular pathways of ferroptosis in CAD and related disorders will enhance our ability to design targeted therapeutic interventions. Second, robust clinical trials are necessary to evaluate the efficacy and safety of ferroptosis-targeting therapies. These trials should also prioritize the development of specific biomarkers to monitor treatment responses and assess drug efficacy in clinical settings. Third, exploring personalized medicine approaches that account for genetic, epigenetic, and environmental factors influencing ferroptosis will enable the development of tailored therapies for individual patients. Lastly, investigating combination therapies that integrate ferroptosis inhibitors with other therapeutic strategies, such as antioxidants or anti-inflammatory agents, may provide synergistic benefits and improve treatment outcomes. Research into these combination approaches should be prioritized to enhance the therapeutic potential of ferroptosis-targeting drugs.

## 7 Summary

CHD, as a complex cardiovascular disorder, involves multiple factors in its pathogenesis. In recent years, the role of ferroptosis as a novel cell death modality in CHD has gradually gained recognition. By reviewing current literature, we have identified that ferroptosis is intimately associated with CHD development, with its mechanisms encompassing multiple aspects including abnormal iron metabolism, lipid peroxidation, and ROS accumulation. Regarding clinical intervention strategies, ferroptosis inhibitors and antioxidants provide promising new therapeutic options for CHD management. However, the precise mechanisms of ferroptosis in CHD have not been fully elucidated, necessitating further in-depth mechanistic investigations and the development of more effective and safer clinical intervention strategies to ultimately improve patient outcomes.
